# Expression of P63, P16 and CK17 in Atypical Squamous Metaplasia and Cervical Intraepithelial Neoplasia

**DOI:** 10.30699/IJP.2021.104280.2095

**Published:** 2020-12-26

**Authors:** Maryam Iranpour, Shahriar Dabiri, Mitra Rezazade-Jabalbarezi, Fatemeh Bagheri

**Affiliations:** 1 *Department of Pathology, Pathology and Stem Cell Research Center, Faculty of Medicine, Kerman University of Medical Sciences, Kerman, Iran*

**Keywords:** Cervical intraepithelial neoplasia, P63, P16, CK17

## Abstract

**Background & Objective:**

Cervical intraepithelial neoplasia (CIN) is a dysmaturation process in squamous cells in epithelial layer, which highly increases the risk of developing cervical cancer. The aim of this study was to compare the expression of three biomarkers, p16, p63, and CK17 in patients with CIN and in those with atypical squamous metaplasia (ASM).

**Methods:**

In this study, 100 patients underwent a colposcopy-guided cervix biopsy. Immunostaining for the biomarkers was undertaken on tissue samples presented with ASM (n=50) and CIN (n=50).

**Results:**

A significant increase in immunostaining for CK7, P63, and P16 in patients with CIN was found compared to ASM subjects.

**Conclusion:**

Expression of CK17, P63, and P16 in CIN varied from those in ASM. Those biomarkers could be reliable factors to distinguish ASM from CIN; however, all the biomarkers could differentiate CIN from its mimics due to their high degree of sensitivity and specificity.

## Introduction

Annually, a total of 330,000 new cervical intraepithelial neoplasia (CIN) cases is reported in the European Union, with relatively half of them diagnosed as CIN I (1). A usually long pre-invasive process capable of developing into invasive cervical carcinoma over time; CIN is microscopically considered as a series of events which progress from cellular atypia to various grades of dysplasia (2).

In accordance with new WHO classification, CIN is categorized into Low-Grade Squamous Intraepithelial Lesion (LSIL) and High-Grade Squamous Intraepithelial Lesion (HSIL) terminology (2). However, based on the degree of dysplasia, previous classifications grouped it into CIN I, CIN II, and CIN III (3). As CIN lesions were monitored and treated in different ways, precise histological grading of CIN lesions assumed of great significance with regard to clinical management of patients. For example, due to its regression in about 80% of cases, CIN I was usually regarded as benign and no therapy was indicated (4). Nevertheless, CIN II and CIN III were considered as precursors to invasive carcinomas and therapy (conization or other less invasive procedures) was indicated since 0.2% to 4.0% of cases with CIN II and CIN III can progress to cervical carcinoma within 12 months (4). It is worth noting that there were no specific clinical symptoms to demonstrate the presence of CIN (5).

In fact, diagnosis of these precursor forms can result in inter-observer variability in comparison with its reactive mimics, such as atypical squamous metaplasia (ASM), immature squamous metaplasia (ISM), reactive/reparative atypia (RA), atrophy, reserve cell/basal cell hyperplasia, etc. Overall, this highlights the need for specific biomarkers to contribute to objective CIN grading and differentiation of true high-grade cervix dysplasia from its mimics (6).

Human papillomavirus (HPV) infection of any type was shown to be related to a 498-fold increase in the risk of cervical cancer development. In comparison to females infected with HPV-16, patients infected with HPV-18 had a higher risk of cervical cancer. The high-risk viruses have a crucial role in the carcinogenic process through production of two oncoproteins encoded by the viral E6 and E7 genes. Directly involved in inactivation of p53 and pRb, respectively, these oncoproteins promote progressive cell cycle and DNA synthesis by blocking apoptosis, thus contributing to viral replication (7).

A tumor suppressor protein, P16 is biologically tasked with regulating cell cycle progression at the G1/S boundary (8), thus demonstration the value of P16 would be as a diagnostic marker for cervical dysplasia and cervical carcinoma (9).

Cytokeratin-17 (CK17), which is an efficient marker for detection of cervical stem cells, is expressed in reserve cells and immature metaplastic cells. However, its expression is not reported in cervical glandular epithelial cells, squamous cells in the portio, or mature squamous metaplastic cells (10).

The areas where P63, a member of the P53 gene family, is expressed include the basal and parabasal cells of mature cervical, vaginal and vulvar squamous epithelium, as well as cervical reserve cells at the transformation zone, immature metaplastic cells, and atrophic cervical squamous epithelium. Instead of Ki67, the present study considers P63 as CIN and squamous metaplasia, e.g., basal cell hyperplasia are mostly the result of basal cell, reserve cell, and stem cell hyperplasia (6).

In order to evaluate the status of HPV infection in cervix, the present study analyzed the most common histological alterations occurring in the cervix, such as squamous metaplasia, CIN 1, CIN 2, CIN 3, as well as expression of the biomarkers of P16, CK17, and P63.

## Materials and Methods

**Patients**

Based on the pathological reports available in the laboratory of Afzalipour Hospital and Besat Clinic, 50 patients with ASM and 50 patients with CIN were enrolled in the study. The study protocol was approved by the Ethics Committee of Kerman University of Medical Sciences, Iran.

**Immunohistochemical (IHC) Staining**

The tissue samples were histopathologically examined by two experts. Formalin-fixed paraffin-embedded tissue was cut in sections with 4μm thickness, which were then placed on slides, dehydrated by alcohol washes, and cleared using a detergent like xylene before being imaged under a microscope. Sections were incubated in 5.0% H_2_O_2_ to quench endogenous peroxidase activity. Furthermore, antigens were also retrieved by digesting the tissue sections with a proteolytic enzyme like trypsin. To decrease the degree of background staining in IHC, the samples were incubated with a buffer, blocking the non-specific sites to which the primary or secondary antibodies may otherwise bind such as normal serum. The slides were then incubated 30 minutes at room temperature with primary antibodies against the antigens including, P16 (rabbit monoclonal), P63, and CK17. In order to detect a biotinylated secondary antibody, the study adopted streptavidin for biotin. To visualize bonding of antibodies, 3, 3μ- diaminobenzidine tetrahydrochloride (DAB) was applied as chromogen for 5 minutes, and the sections were counterstained in hematoxylin for 2 minutes.

**Evaluation of P16, CK17 and P63 Expression**

The immunoreactivity of P16 and P63 was shown to be positive since more than 50% and 10% of the tumor cell nuclei indicated a strong intensity, respectively. The P16 was considered as positive when it showed nuclear and continuous cells diffuse cytoplasmic staining in the basal and para-basal squamous epithelium cell layers, which variably reached intermediate and superficial cell layer characteristic of a diffuse staining pattern. Additionally, P16 was shown to be negative when it was completely unstained or demonstrated a focal or sporadic epithelial staining, not characterized by basal and para-basal cells (focal staining pattern). CK17 staining indicated to be positive when cytoplasmic staining involved all squamous cell layers. Furthermore, focal staining or completely unstained cell layers were considered as negative.

**Statistical Analysis**

The data were analyzed by IBM SPSS Statistics for Windows, version 20 (IBM Corp., Armonk, N.Y., USA) using the statistical methods such as Chi square test and screening test. The statistical significance was considered to be 0.05.

## Results

**Fig. 1:**
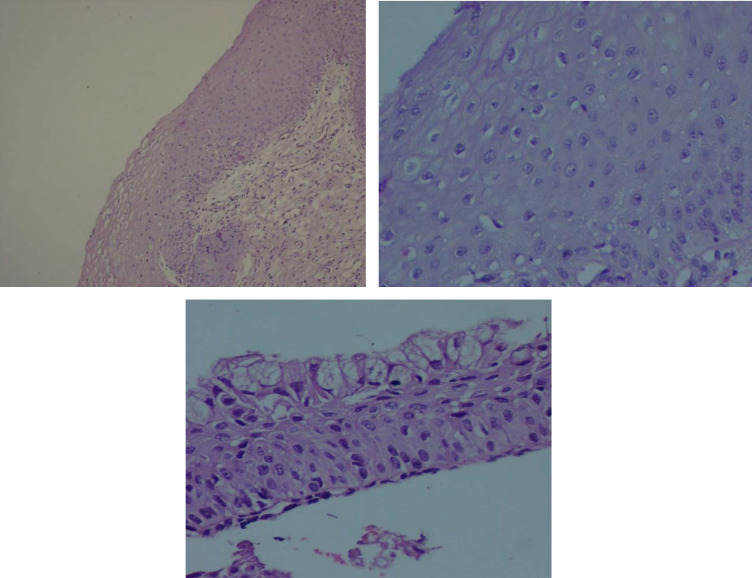
H&E Staining, Moderate Dysplasia. A) ×100; B) ×400; C) Squamous metaplasia

Out of 100 patients included in the study, 59 and 41 subjects were diagnosed with CIN and ASM, respectively. Considering an appropriate staining, the samples were enrolled for interpretation based on the distribution, localization, and pattern of involvement. Figures 1-A and 1-B show exocervical tissue with nuclear elongation and dysmaturation of squamous epithelial cells in the lower two thirds of exocervical tissue and Figure 1-C shows squamous metaplasia. 

**P63 Expression**

**Fig. 2:**
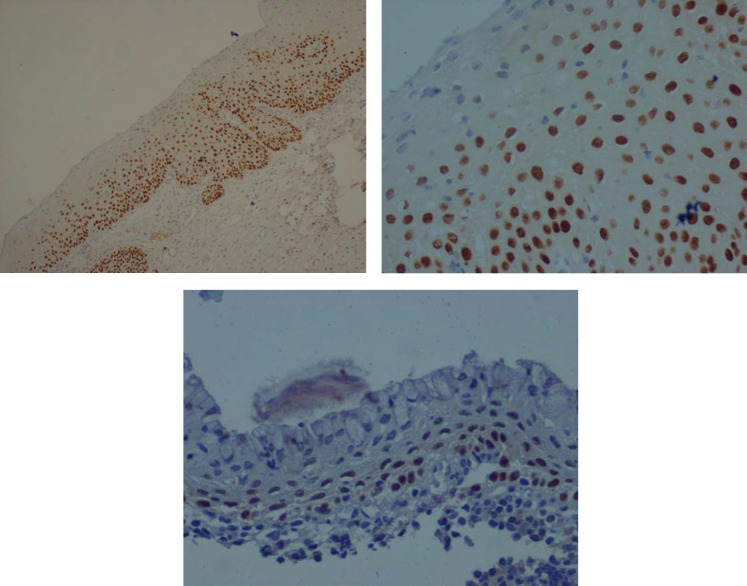
Positive nuclear staining of P63 in dysplastic cells. A) ×100; B) × 400; C) Negative staining of nuclear P63 in squamous metaplastic cells

In ASM, 8 (19.5%) cases were positive for P63 (Table 1). In the group with ASM and high grade CIN, 61/100 patients were positive for P63. In the high grade CIN group, 53 (89.8%) subjects showed positive P63 (Table 2). Moreover, P63 was found to be a statistically significant marker (*P*<000) with regard to differentiating CIN from ASM. Figures 2-A and 2-B show nuclear staining of P63, indicating a positive reaction in atypical squamous epithelial cells in the lower two thirds of exocervical tissue, and Figure 2-C shows nuclear staining of P63 indicating a negative reaction in squamous metaplastic cells.

**Table 1:** P63 Staining in Atypical Squamous Metaplasia

Tissue	P63	Frequency	Percent
Metaplasia	Positive	8	19.5 %
	Negative	33	80.5 %
	Total	41	100 %

**Table 2:** P63 Staining in High Grade CIN

Tissue	P63	Frequency	Percent
Dysplasia	Positive	53	89.8 %
	Negative	6	10.2 %
	Total	59	100 %

Abbreviations: CIN, Cervical Intraepithelial Neoplasia.

**Fig. 3:**
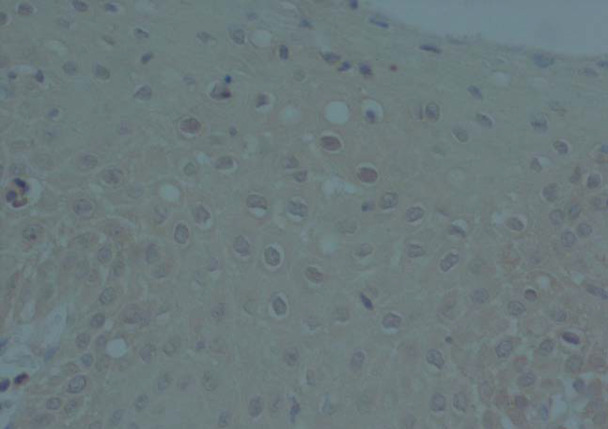
Immunohistochemical Staining of P16

**P16 Expression**

P16 was shown to be positive in 30% of all cases. Moreover, 30 (50.8%) CIN cases showed positivity whereas No positivity for P16 was found in ASM and squamous metaplasia (100%), while 50.8% of the cases of dysplasia showed positive reactivity for that marker (Tables 3 and 4). According to the findings, there was a strong relationship between P16 expression and progression of the lesion with a P-value<0.00. Figure 3 demonstrates immunoreactivity of P16 that was negative in basal and atypical squamous epithelial cells.

**Table 3:** P16 Staining in Atypical Squamous Metaplasia

Tissue	P16	Frequency	Percent
Metaplasia	Positive	0	0
	Negative	41	100 %
	Total	41	100 %

**Table 4:** P16 Staining In High Grade CIN

Tissue	P16	Frequency	Percent
Dysplasia	Positive	30	50.8 %
	Negative	29	49.2 %
	Total	59	100 %

Abbreviations: CIN, Cervical Intraepithelial Neoplasia.

**CK17 Expression**

**Fig. 4:**
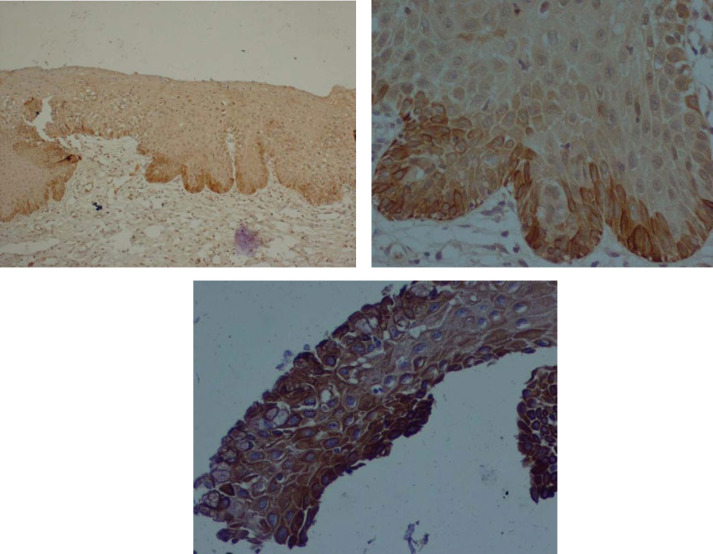
Cytoplasmic Staining of CK17 in basal layer. A) × 100; B) ×400; C) Positive cytoplasmic staining of CK17 in squamous metaplastic cells

CK17 was found to be positive in 43% of all cases. CIN showed 8.5% positivity whereas ASM showed 92.7% positivity (Tables 5 and 6). CK17 was statistically insignificant (*P*=0.0) in terms of distinguishing CIN from ASM. Figures 4-A and 4-B show positive immunoreactivity of CK17 only in basal layer, which was negative in atypical squamous, and Figure 4-C shows positive cytoplasmic of CK17 in squamous metaplastic cells. P63 was found to be positive in 50.28% of all the case that 89.8% were diagnosed as dysplasia and 19.5% showed metaplasia. P16 was found to be positive in 29.78% of all the cases, of which 50.8% diagnosed as dysplasia. CK17 was found to be positive in 69.8% of all the case of which 8.5% showed dysplasia and 92.7% demonstrated metaplasia (Table 7)

**Table 5:** CK17 Staining In Atypical Squamous Metaplasia

Tissue	CK17	Frequency	Percent
Metaplasia	Positive	38	92.7 %
	Negative	3	7.3 %
	Total	41	100 %

Abbreviations: CK17, cytokeratin17.

**Table 6:** CK17 Staining In High Grade CIN

Tissue	CK17	Frequency	Percent
Dysplasia	Positive	5	8.5 %
	Negative	54	91.5 %
	Total	59	100 %

Abbreviations: CIN, Cervical Intraepithelial Neoplasia; CK17, Cytokeratin17.

**Table 7:** Summary of Immunohistochemical Staining Profile, Atypical Squamous Metaplasia and Cervical Intraepithelial Neoplasia (CIN).

IHC Markers	Diagnosis	Positive	Negative	Pearson Chi- Square	DF	Asymp. Sig. (2-sided)
P63	Dysplasia	89.8 %	10.2 %	50.28	1	.000
	Metaplasia	19.5 %	80.5 %			
P16	Dysplasia	50.8 %	49.2 %	29.78	1	.000
	Metaplasia	0 %	100 %			
CK17	Dysplasia	8.5 %	91.5 %	69.98	1	.000
	Metaplasia	92.7 %	7.3 %			

Abbreviations: CIN, Cervical Intraepithelial Neoplasia; CK17, Cytokeratin 17; IHC, Immunohistochemistry.

## Discussion

Cervical cancer is the first cause of cancer in females aged 15-45 years old, suggesting that the main etiology is the young age for the onset of sexual intercourse. In this study, IHC staining was used to examine the expression of P16, P63, and CK17 to distinguish high grade CIN lesions from ASM. According to the research results, P63 expression was found to be 89.8% and 19.5% in dysplasia and metaplasia, respectively. Furthermore, the expression of P63 in CIN was increased significantly compared to metaplasia. However, it is still unknown whether P63 is a tumor suppressor gene or an oncogene (11). On the other hand, applying P63 in metastatic and primary tumors is controversial (9, 12, and 13). Findings of the present study revealed that P63 significantly increased in dysplasia lesions. The expression of P63 gene in tumor lesions has also been reported in a number of other studies whose results were similar to those of ours. However, the results of few studies were inconsistent with our findings (5, 13, and 14). The present study supports the findings of Regauer S & Reich O (2007), which suggested the increase in marker P63 was directly correlated with the degree of labelling and the degree of dysplasia (15). In the study by Selvi *et al.* (2014), P63 expression reported in 343/350 cases (98%) showed positivity, while it was negative in 7/350 cases (2%) (6). This pattern of positivity is comparable with a few previous studies, including Jolise (2004) and Quade (2001) that analyzed P63 expression in CIN; they concluded that P63 expression has an increasing trend in high-grade CIN (14, 16). In addition, it has been shown that P63 can be utilized to make a distinction between a proliferating epithelium (CIN, ISM, and basal cell hyperplasia) and normal/non-proliferating epithelium (RA) (6).

With regard to findings of the present study, P16 staining of metaplasia samples is 100% negative. Meanwhile, it was also shown that the presence of P16 was positive in 50.8% of the dysplasia samples. As a cyclin-dependent kinase inhibitor, P16 acts as a tumor suppressor (17). Recent studies have shown some neoplasia, including cervical cancer where P16 is overexpressed (18-20). The effect of feedbacks capable of suppressing the tumor accounts for the increase in this marker during epithelial damage (15).

The research carried out by Regauer S & Reich O (2007) concluded that immature metaplasia had consistent characteristics like strong, uniform CK17 staining of the proliferating cells alongside P16 negativity, whereas high-grade dysplasias⁄CIN III revealed a mirror image IHC profile characterized by strong diffuse staining of all dysplastic proliferating cells with P16 (15). Furthermore, the study by Xing *et al.* (2017) stated that P16 marker might be a biomarker contributing to making a distinction between CIN1 and CIN2/3 (21). Cervical lesions are shown to be generally associated with positive P16, though the results vary from one study to another, depending on the degree of lesions and immunity. The highest level of heterogeneity is seen in CIN I category, while P16 expression ranges from 35% (22) to 100% (23). Although P16 was reportedly positive in more than 90% and up to 100% in CIN II and CIN III grades (23, 24), a high negative ratio (up to 33%) has been also reported (22). In many cases, P16 can help distinguish CIN from similar cases, hence being an efficient factor in accurate diagnosis and proper management of patients in addition to histomorphology, as recommended by many studies (25, 26).

With regard to CK17, this marker showed an overall positivity of 97% in squamous metaplasia, even though its expression in dysplasia tissue declined by 8.5%, which was statistically significant.

Regauer S & Reich O (2007) maintained that metaplasia showed a strong expression for CK17 marker while dysplasia was negative for CK17; they suggested that CK17 marker can be employed in IHC method to differentiate metaplasia from dysplasia, which is a finding in line with that of the present study (15). Coinciding with results of this study, the findings obtained by Sari Aslani *et al.* (2013) showed that IHC staining for CK17 can be positive in metaplasia, and in some CIN lesions (27). Also, Selvi *et al.* (2014) concluded that CK17 has varied positivity in both CIN and the benign mimics, and that positivity is the result of more staining. They stated that, contrary to this study, HIC is useful for distinguishing different degrees of CIN from each other and from its benign mimics (6). In another study, Escobar-Hoyos *et al.* (2013) reported that CK17 can have a diagnostic utility for cervical squamous metaplasia (28), a finding supporting our evidence. Nonetheless, both studies by Smedts *et al.* do not agree with viewing CK17 marker as a diagnostic utility for squamous metaplasia, claiming that CIN is positive only in reserve cells and in varying degrees (29, 30). This result is contrary to that of the present study. Differences experienced in various studies may be due to the fact that CIN cases in Iran are not common compared to other countries.

## Conclusion

When difficulties are encountered with histomorphology to make a definite and accurate diagnosis of CIN, a combination of P16, P63, and CK17 markers would be useful for distinguishing CIN from its mimics. Further studies are needed, especially on other markers.
